# Mapping the experiences of adult cancer patients receiving chimeric antigen receptor T-cell (CAR-T) therapy: A scoping review protocol

**DOI:** 10.1371/journal.pone.0338210

**Published:** 2025-12-05

**Authors:** Isabela Girleanu, Anne-Marie Brady, Vanessa Boland

**Affiliations:** Faculty of Health Sciences, Trinity Centre for Practice & Healthcare Innovation, School of Nursing and Midwifery, Trinity College Dublin , Dublin, Ireland; University of California Santa Barbara, UNITED STATES OF AMERICA

## Abstract

**Introduction:**

Chimeric antigen receptor T-cell (CAR T) therapy is an advancement in cancer treatments, particularly for patients with haematological malignancies who have exhausted conventional therapies like chemotherapy and stem cell transplant. CAR-T therapy involves genetically engineering a patient’s own T-cells to attack cancer cells. The development of CAR-T therapy increased survival rates in 30% to 80% of patients. Due to its novelty, CAR-T therapy can present unforeseen challenges that prolong the experience of living with cancer and its treatment journey. While CAR-T cell therapies have the potential to be curative, these innovative treatments are often accompanied by a significant symptom burden, primarily cytokine release syndrome (CRS) and immune effector cell associated neurotoxicity (ICANS).

**Methods and Analysis:**

The Arksey and O’Malley framework and the population, concept and context (PCC) framework will guide the development of this scoping review. The population of this review is adults (18 years of age and older). The concept scoped is experiences of patients in the context of CAR-T therapy. CINAHL, Medline (EBSCO), PsycINFO, EMBASE and Web of Science will be searched, including grey literature. Screening and data extraction will be conducted by two independent researchers following the inclusion criteria and The Preferred Reporting Items for Systematic Review and Meta-Analysis Protocols (PRISMA-P) checklist. The protocol has been registered on Open Science Framework on 13^th^ of May 2025.

**Study Status:**

Record screening is currently ongoing and it is estimated to be completed by 21^st^ of July 2025. Data extraction is estimated to commence on 22^nd^ of July 2025 and to be completed by 4^th^ of August 2025. Results are to be expected on 15^th^ of August 2025.

**Dissemination of Findings:**

Findings will be disseminated to relevant healthcare services electronically, through oral or poster presentations, conferences, congresses and publications.

## Introduction

Chimeric antigen receptor T-cell (CAR-T) therapy has been recently developed to treat haematological malignancies [1,2]. CAR-T therapy involves the removal of a patient’s own T-cells and genetically engineering them to attack cancer cells [3]. CAR-T therapy is available for subtypes of lymphoma, leukaemia and myeloma [4]. To qualify, the patient must have undergone and failed at least two rounds of treatment such as chemotherapy and/or stem cell transplantation; for cancer that is either refractory (cancer cells do not respond to treatment) or has relapsed (cancer cells started to grow again after a period of remission) [2].

CAR-T therapy can be potentially dangerous largely due to complex life-threatening complications [5]. One of the most significant complications of CAR-T therapy is cytokine release syndrome (CRS), a whole-body allergic response to the re-infused cells [6,7]. CRS is estimated to occur in 57% to 93% of patients and can lead to headaches, diarrhoea, nausea, vomiting, inflamed lymph nodes, rigors, fever, hypotension, hypoxia, liver dysfunction and renal impairment [8]. Almost half of patients who suffer from CRS require intensive care management [9]. The second major complication is a brain-specific immune response to the re-infused cells, known as immune effector cell-associated neurotoxicity syndrome (ICANS) [10,11]. ICANS is estimated to occur in 39% to 69% of patients and can lead to severe confusion, changes in speech, coordination, handwriting, seizures and even cerebral oedema [12]. Notably 85% of all patients who suffered from ICANS require intensive care management [13].

The development of CAR-T therapy has increased the survival rates of 30% to 80% of patients who have exhausted conventional therapy methods [4]. Due to its novelty, CAR-T therapy can present unforeseen challenges that prolong the experience of living with cancer and its treatment journey [14,15]. Innovative cancer treatments like CAR-T therapy can be complex resulting in a preparation, administration and recovery process that can be burdensome to patients [16,17]. Limited evidence on patient experiences of CAR-T therapy are available; this scoping review aims to address this knowledge gap [18].

## Design, methods and analysis

The Arksey and O’Malley [19] framework for scoping review studies will be used following these five stages.

### Stage 1: Identifying the scoping review question

The population, concept and context (PCC) framework was used to frame the scoping review question.

- Population: Adult cancer patients who have been treated with chimeric antigen receptor T cell (CAR-T) therapy for haematological malignancies.
- Concept: The experiences relating to chimeric antigen receptor T-cell (CAR-T) therapy.
- Context: Chimeric antigen receptor T-cell (CAR-T) therapy.

#### Research question.

1. What are the experiences of patients receiving chimeric antigen receptor t-cell (CAR-T) therapy?

#### Objectives.

1. To evaluate patients’ experiences of CAR-T therapy.
2. To evaluate the symptoms and quality of care outcomes of patients undergoing or who have undergone CAR-T therapy.
3. To inform the development and care of patients receiving CAR-T therapy.

### Stage 2: Identifying relevant studies

#### Search strategy.

This scoping review will follow a three-step search strategy as advised by Hadie [20]. First, an initial search will be conducted of two databases, CINAHL and PsycINFO. Titles, abstracts, and the index terms from the retrieved papers will be analysed to identify relevant key words. Guided by the PCC framework these keywords will be expanded upon using a thesaurus to ensure comprehensive coverage. Next, a search will be conducted across all databases using the refined index terms and keywords. The databases included will be CINAHL, Medline (EBSCO), PsycINFO, EMBASE and Web of Science. The reference list of identified articles will be reviewed to uncover additional sources. Grey literature will also be searched and if necessary, authors will be contacted for clarification or further information. Finally, an expert university librarian/information retrieval specialist will aid in designing and refining the search strategy. Another team member will peer review the search strategy. A search strategy for at least one major database is included in Table 1.

**Table 1:** Sample search terms for the CINAHL database.

PCC concept	Initial keywords
Population Adult cancer patients.	((cancer* OR oncolog* OR malign* OR haemo-oncol* OR hemo-oncol* OR neoplasm* OR haemotol* OR hemotol*) N3 (patient* OR survivor* OR individual* OR person* OR people* OR client* OR adult*))
Concept Experiences (relating to qualitative research & research types)	qualitativ* OR experien* OR view* OR viewpoint* OR percept* OR perceiv* OR attitud* OR belief* OR perspectiv* OR opinion* OR thought* OR awar* OR comprehen* OR understand* OR feel* OR challeng* OR difficult* OR obstacl* OR need* OR unmet* OR narrativ* OR “action research” OR “case stud*” OR descript* OR describ*
Context Chimeric antigen receptor t-cell (CAR-T) therapy.	(“chimeric antigen receptor*” OR “car t” OR “car-t” OR axicabtagene OR brexucabtagene OR lisocabtagene OR tisagenlecleucel) N2 (therap* OR treat* OR manag* OR administ* OR support*)

#### Inclusion and exclusion criteria.

This scoping review will include primary and empirical studies, encompassing longitudinal and cross-sectional studies, as well as qualitative and quantitative studies, and mixed methods studies that report on the experiences of adult patients undergoing chimeric antigen receptor T-cell (CAR-T) therapy. As outlined in Table 2, no search restrictions will be applied. Unpublished grey literature will also be considered.

**Table 2:** Inclusion and exclusion criteria.

*Criterion*	*Inclusion*	*Exclusion*
*Language*	Any language	None
*Focus*	Articles that focus on the experiences of adults (age 18 years and over) who are receiving or have received CAR-T therapy.	Articles that do not focus on the outcome of interest (experiences including symptom experiences, quality of life) or the wrong population (under 18 years of age).
*Time period*	Any time period	None
*Types of articles*	Primary/empirical studies, longitudinal and cross-sectional studies, qualitative and quantitative studies, and mixed methods studies. Conference submission and abstracts. Unpublished grey literature including theses, dissertations, government/industry reports book chapters.	Articles with insufficient details to extract the experiences of CAR-T therapy from an adult patient perspective.

### Stage 3: Source of evidence selection

Two independent reviewers will screen by title and abstract using the inclusion and exclusion criteria. This will be followed by a full text screen. Any discrepancies between the two reviewers regarding the inclusion and exclusion of a source will be resolved by a third reviewer to achieve consensus. Sources will be screened using Covidence. Before screening begins, a pilot screening test of 25 randomly selected titles/abstracts will be conducted using the inclusion and exclusion criteria. Further refinement of criteria will occur as required. Duplicates will be automatically removed when sources are imported into Covidence. Screening is currently ongoing and it is estimated to be completed by 21^st^ of July 2025. The study selection process will be reported following the Preferred Reporting Items for Systematic Review and Meta-Analysis extension for Scoping Reviews Protocols (S1 File) checklist [21].

### Stage 4: Data extraction

Two reviewers will carry out a pilot test of the data extraction table (S1 Appendix) using a 10% sample of the complete list of retrieved studies to be included in the scoping review. Discrepancies arising between the two reviewers will be resolved by consulting with the third reviewer. This will ensure transparency throughout the data extraction methods. Any required modifications to the categories of the data extraction table can be integrated at the end as required. Data extraction is estimated to commence on 22^nd^ of July 2025 and to be completed by 4^th^ of August 2025.

### Stage 5: Collating, summarising and reporting the results

Data analysis will involve descriptive quantitative analysis and qualitative thematic analysis. The data extraction table (S1 Appendix) will be used to aid data extraction. The extracted data will be presented in tabular and graphic form. A narrative summary will accompany the tabulated results, describing how the study’s findings relate to the review’s objectives and research question. The summary will clearly outline the purpose and methodology of each study and ensure its alignment with the scoping review aim and objectives. The results are to be expected on 15^th^ of August 2025.

#### Assessment of methodological quality.

Using critical appraisal tools will assist in assessing the trustworthiness, relevance and results of published papers included in this scoping review. Hence, to ensure that only high-quality studies are included, The Joanna Briggs Institute [22] critical appraisal tools will be used.

#### Ethics and dissemination.

This scoping review does not require ethical approval. The findings will be disseminated to relevant healthcare services electronically, through oral or poster presentations, conferences, congresses and publications.

#### Study status.

The final protocol was registered prospectively with the Open Science Framework on 13^th^ of May 2025. Initial searches has commenced and author roles had been delegated.

## Discussion

A preliminary search of current evidence conducted as part of this scoping review protocol revealed a significant gap in research on the patient experiences of CAR-T therapy. Specifically, there is a lack of studies exploring of adult patients’ experiencing this nuanced and complex cancer therapy. To enhance patient outcomes and delivery of care research that adopts a more person-centred approach is needed to better understand the complexities of patients’ experiences and their unique needs

A scoping review is the first step in mapping the current evidence on this topic, and it aids in grasping the depth and breadth of the information available in each relevant source [18,20]. This scoping review will answer the following research question: ‘What are the experiences of patients receiving chimeric antigen receptor t-cell (CAR-T) therapy?’ and will achieve the following objectives: will evaluate the extent to which patients’ experiences has been researched about CAR-T therapy; will evaluate the symptom and quality of care outcomes of patients undergoing or who have undergone CAR-T therapy and will inform development and care of patients concerning CAR-T therapy. A scoping review is limited in addressing the feasibility, appropriateness, meaningfulness or effectiveness of a certain treatment or practice [18]. Therefore, a limitation of this systematic review is providing concrete guidance from a policy-making point of view [20].

We anticipate that the results of this scoping review will not only contribute to existing literature but also inform policy guidelines aimed at improving and enhancing care for CAR-T patients. The findings of this scoping review will be disseminated to relevant healthcare services electronically, through oral or poster presentations, and shared at conferences, congresses and publications.

## Supporting information

S1 File PRISMA-P-checklist. (PDF)

S2 File PRISMA-ScR. (PDF)

S1 Appendix Data extraction table. (DOCX)
