# Supplementary material for: Mapping the experiences of adult cancer patients receiving chimeric antigen receptor T-cell (CAR-T) therapy: A scoping review protocol
Source: PLoS One. 2025 Dec 5;20(12):e0338210. doi: 10.1371/journal.pone.0338210 (PMC12680201; doi:10.1371/journal.pone.0338210)
Supplement: S1 Appendix — (DOCX) [file pone.0338210.s003.docx]

Appendix 1: Data Extraction Table

| Author | Year | Title | Country or Origin | Aim | Method | Instruments | Recruitment | Participants | % Male | % Female | Age Range | % Lymphoma | % Other Cancers | Time Since Diagnosis | Main Treatment | Time Since Treatment | Prior Lines of Treatment | Ethnicity/Race | % Employed | Education | % Partnered | Relevant Information |
| --- | --- | --- | --- | --- | --- | --- | --- | --- | --- | --- | --- | --- | --- | --- | --- | --- | --- | --- | --- | --- | --- | --- |
|  |  |  |  |  |  |  |  |  |  |  |  |  |  |  |  |  |  |  |  |  |  |  |
